# A Just Tribute

**Published:** 1868-02

**Authors:** 


### Selections.

### A JUST TRIBUTE.

It affords us much pleasure to give place to the following from an exchange; and we feel confident that any true-lover of our profession will feel gratified, if not proud of this recognition of the triumphs of the Dental profession in this country. This has not been an easy thing to attain, as many know; and its accomplishment places the profession under an additional debt of gratitude to Dr. Allen, which, we trust its members will not be slow to discharge.—Ed.

The improvements of Dentists, physicians and surgeons were differently classified, and the awards of merit granted to them were designated by official reports from the imperial commission, while the awards of the artisans and manufacturers were represented simply by medals. A report on Class xi, Group ii, Paris Exposition, 1867.

The jury on dentistry at the Champs de Mars have reported that "The specimens of 'Continuous gum sets of teeth, upon platinum plate,' by John Allen & Son, of New York, are incomparably the most beautiful pieces exhibted." This report has been corroborated by various correspondents writing from Paris.

The New York *Times*, of May 14th, 1867, has an article from its correspondent, saying of American Dentistry, "It is beyond all competition. The display of artificial teeth, the perfect imitation of nature, in gums, and palate, or roof, are wonderful. But the French are equally great in eyes and ears."

A Philadelphia correspondent, writing from Paris, April 15th, 1867, says: "The case of teeth by J. Allen & Son attracts much attention. It is very much admired. Nothing like them seems to be known here; and people ask all sorts of questions regarding them. They are far in advance of the times here."

A New York correspondent writes: "Paris, June 12th, 1867. I have conversed with many persons here on the subject of Dentistry on exhibition, and they all agree that the Artificial Dentures of J. Allen & Son surpass any and everything of the kind here represented."

A Parisian dentist writes to a friend in New York, July 13th, 1867: "I embrace this opportunity of testifying to the excellence and superiority, above all others of the dental work here of Allen & Son." Another Parisian dentist writes: "You may feel sure there is nothing in the whole exhibition of dentistry which can in any way approach the perfection and beauty of the dental work of Allen & Son."

The above official report from the old world is re-echoed by the American Institute at their great Fair, held in New York City, during October, 1867, when another medal was awarded J. Allen & Son, together with the following report from the Judges of Dentistry: "Case No. 508, Mounted Artificial Teeth on Platinum Base, by J. Allen & Son, No. 22 Bond street, New York City.

THE BEST ON EXHIBITION.

Their merits are strength, durability, cleanliness, and adaptation to every conceivable physiognomical requirement of the teeth, and color of the gums."

